# Estimating the Cost and Payment for Sanitation in the Informal Settlements of Kisumu, Kenya: A Cross Sectional Study

**DOI:** 10.3390/ijerph14010049

**Published:** 2017-01-06

**Authors:** Sheillah Simiyu, Mark Swilling, Richard Rheingans, Sandy Cairncross

**Affiliations:** 1 School of Public Leadership, Stellenbosch University, Stellenbosch 7602, South Africa; Mark.Swilling@spl.sun.ac.za; 2 Community Health Department, Great Lakes University of Kisumu, Box 2224-40100, Kisumu, Kenya; 3 Sustainable Development Department, Appalachian State University, Boone, NC 28608, USA; rheingansrd@appstate.edu; 4 Department of Environmental and Global Health, University of Florida, Gainesville, FL 32611, USA; 5 Department of Disease Control, London School of Hygiene and Tropical Medicine, Keppel Street, London WC1E 7HT, UK; Sandy.Cairncross@lshtm.ac.uk

**Keywords:** sanitation, landlord, socio-economic, hedonic pricing, housing, tenant

## Abstract

Lack of sanitation facilities is a common occurrence in informal settlements that are common in most developing countries. One challenge with sanitation provision in these settlements is the cost and financing of sanitation. This study aimed at estimating the cost of sanitation, and investigating the social and economic dynamics within Kisumu’s informal settlements that hinder provision and uptake of sanitation facilities. Primary data was collected from residents of the settlements, and using logistic and hedonic regression analysis, we identify characteristics of residents with sanitation facilities, and estimate the cost of sanitation as revealed in rental prices. Our study finds that sanitation constitutes approximately 54% of the rent paid in the settlements; and dynamics such as landlords and tenants preferences, and sharing of sanitation facilities influence provision and payment for sanitation. This study contributes to general development by estimating the cost of sanitation, and further identifies barriers and opportunities for improvement including the interplay between landlords and tenants. Provision of sanitation in informal settlements is intertwined in social and economic dynamics, and development approaches should target both landlords and tenants, while also engaging various stakeholders to work together to identify affordable and appropriate sanitation technologies.

## 1. Introduction

Informal settlements are faced with a number of challenges, including insecure land tenure, poverty, overcrowding and a lack of basic services and infrastructure [1,2,3,4,5,6]. Sanitation is one of the basic services often lacking in informal settlements, a situation attributed to various factors, including limited public finances at the governmental level [7] and a reluctance from local governments to allocate public funds for such private goods as sanitation [8]. As a result, sanitation has been accorded a low financing priority in informal settlements. Nonetheless, it is important to estimate the cost of sanitation in order to design appropriate financing and cost recovery strategies, to determine if subsidies are required, and establish the kind of subsidies required [9]. Since subsidies may be costly, private/self-financing options are financing alternatives that can be explored [9]. Households in informal settlements often provide their own sanitation facilities [10,11]. Such self-provision in poor urban areas often implies that households have to purchase basic services (including sanitation), just like they purchase other commodities [12]. It thus becomes imperative to identify who pays for sanitation, how much they pay, what they pay for, and how they pay for it [13], information that is important for overall development of informal settlements.

Drawing from environmental economics, it is possible to estimate the cost of goods and services through information given by respondents about their preferences (stated preference), or through observation of behaviour (revealed preference). Stated preference methods have faced several critiques, including challenges of reliability and validity [14,15,16,17,18,19,20], since they are not based on what people actually do [21]. In addition, due to the hypothetical nature of most willingness to pay studies, it is argued that respondents may be ignorant, uncertain or unable to make a trade-off on the good or service [16,22,23]. Revealed preference methods, however, are based on actual behaviour [24]. The Hedonic Pricing Method (HPM), which is a revealed preference approach, is largely used in the real estate market, and it estimates the willingness to pay for characteristics or services (for instance of a house) as reflected in purchase or rental prices [25,26,27]. It analyses effective demand in comparison to the projected perceptions of demand in stated preference methods [28,29]. Since the hedonic method is based on actual behaviour and decisions that people have made rather than assessments of hypothetical alternatives from which their willingness to pay is deduced [24,30], it is said to have high content validity [30].

This economic background can be used to estimate cost of sanitation in informal settlements, but it is important to understand the complexities in informal settlements that affect sanitation provision. Studies from various informal settlements such as those in Kenya [31], Senegal [32], Lesotho and Mozambique [33,34], reveal that a majority of residents in informal settlements are tenants. Most tenants are less motivated to invest directly in sanitation facilities, as they consider it the land owner’s responsibility [8,35,36]. In addition, most tenants may not know the cost of sanitation as noted in Uganda’s informal settlements [37]. It is likely, therefore, that tenants may under- or over-estimate the amounts that they are willing and/or able to pay for sanitation through stated preference methods. On the other hand, research [35,38] suggests that tenants may pay for sanitation if the costs are indirectly included in their house rental prices. Therefore, in order to determine the cost of sanitation in informal settlements, hedonic pricing method can be used to estimate how much tenants are indirectly paying for sanitation through house rental prices, and how the dynamics in informal settlements influence payment for and provision of sanitation. This study therefore takes on a hedonic approach to estimate the cost of sanitation as revealed through house rental prices in Kisumu’s informal settlements. A summary of the hedonic pricing method will be presented, followed by a description of the study area and the methods used for data collection and analysis. Results, a discussion and a conclusion then follow, with the paper providing some policy implications. The main conclusion of this study is that provision of sanitation in informal settlements is crucial and requires the involvement of the necessary stakeholders, including landlords.

## 2. The Hedonic Pricing Method (HPM)

The theory behind HPM is that the selling or rental price of a house depends on the buyers’ preference for the characteristics of the house. The property is assumed to be sold in a perfectly competitive market and therefore, the buyer determines the price he pays by choosing his preferred attributes [30,39]. The price paid for the property is therefore a function of the attributes [30], and even though consumers pay a bundled price for the house, they are essentially paying for the individual attributes [40]. The hedonic method is thus used to evaluate the willingness to pay for these attributes [26,27,41].

The equation of the hedonic price function is presented as:

P*_i_* = *f* (x*_i_*;β) + *u_i_* (1)

where P*_i_* is the selling price, x*_i_* are attributes of the house (which include characteristics such as number of rooms, and access to neighbourhood services such as schools and workplaces), β is the vector of coefficients, and *u_i_* represents the part of the price that is non-explained [25,42].

The relationship described in Equation (1), between the price and the attributes, is a linear model; although it can take other forms such as the semi-log, double log, quadratic, and box cox models [21,39,43]. The linear model, just like the normal linear regression model, assumes that the relationship between the dependent variable (house price or rent) and the other independent variables is linear. It is faulted for the assumption it makes that the price of the independent variables is constant, which is not always the case in the real market [39,43]. In a semi-log model, the independent variables remain untransformed, while the dependent variable takes on the natural log form. A unit change in the independent variable leads to a certain percentage change in the dependent variable. In a double log model, both the dependent and independent variables are transformed to the natural log form, implying that a percentage increase in the dependent variable is due to a percentage increase in the independent variable. The box-cox transformation model encompasses the three models through a box-cox transformation [43,44]. It is important that the relationship between the price and the key characteristics of the study are understood [39] so that the best model is selected, which should be one that gives the best estimates/fit and explanation that is based on the data [29,43].

Few studies have applied the HPM to estimate the cost of sanitation in informal settlements, and the willingness of the urban poor residents to pay for sanitation. These studies include those carried out by Gulyani et al. [45] in Nairobi (Kenya) and Dakar (Senegal) and that by Brueckner [46] in Indonesia. This study therefore provides insight on the cost of and willingness to pay for sanitation in the informal settlements of one of the rapidly expanding cities in Kenya-Kisumu city.

## 3. Study Area and Methods

### 3.1. Study Area

Kisumu city is in Kisumu County and is the third largest city in Kenya, with a population of approximately 420,000 [47]. More than half of the city’s population is poor [48], and it is estimated to have one of the highest proportion of its residents, approximately 60% of the population [49], living in informal settlements [50]. These settlements have characteristics such as poor housing units, a lack of sanitation facilities, and poor waste disposal [48].

Residents commonly live in plots/compounds, which is a group of housing units that have been constructed by one landlord but are occupied by different households. Most of the inhabitants are tenants, although some landlords also live within their compound [51]. Landlords may also live elsewhere and not within their compounds with the tenants, thus being absentee landlords. Within the compounds, households share amenities such as water and sanitation [51].

In terms of sanitation, the sewer system does not serve the informal settlements; and the most dominant sanitation facilities are traditional pit latrines and a few septic tanks [52]. It is estimated that half of the compounds in the settlements lack sanitation facilities, with “flying toilets” (the practice of defecating in a plastic bag and flinging it away) being a common practice [51]. This lack of sanitation is worsened by conditions such as high water tables, loose soils and flooding during the rainy season, which have led to the collapse of pit latrines in the settlements [48,53].

### 3.2. Sampling and Data Collection

This study adopted a cross sectional design, in which data was collected from the informal settlements between January and June 2014. Preliminary study findings (i.e., expected difference (0.27), standard deviation (0.48) at a 90% statistical power and the 95% confidence level, while adjusting for a 20% non-response rate) were used to calculate the required sample size of 160 compounds.

Data was collected from the Nyalenda A, Nyalenda B, Bandani and Obunga settlements. Because of a lack of data on the number of compounds in each of the informal settlements, the sample size was divided equally among the four settlements ([54], pp. 175–176), thus 40 compounds from each settlement.

These settlements are further divided into units, which are geographical regions in the settlements, commonly used for subdivision and identification. For example, Nyalenda B, is divided into Kilo, Dunga, Nanga, Got Owak and Western units [55]. Two units were purposively selected from each settlement. This selection of units was based on population density and settlement patterns (selected units were those that had high population density and rent-paying tenants). Twenty compounds were assigned to each unit. Again, because of a lack of data on the number of compounds in each unit, transect walks were carried out in the selected units to determine the approximate number of compounds. This approximate number was then divided by the desired sample size from each unit, in order to determine the sampling interval. On average the sampling interval was three compounds.

Selection of compounds began from one end of the unit towards the other end, by systematically skipping the determined sampling interval. Data collection was carried out by research assistants who worked in two groups of two people. One group began from one end of each unit and another group began from another end of the unit. Upon arrival at a compound, research assistants first verified if there were rent paying residents within the compound. If the compound lacked tenants, the assistants moved to the next compound. If there were tenants within the compound, assistants randomly selected one household from the households in the compound. An adult household head or their spouse from the selected household was then selected for interviewing. Research assistants explained to the selected respondent the purpose of the research, and their roles and rights as respondents. They were given time to consent to participating, and after granting consent, the interview began.

One research assistant interviewed the respondent, while the other completed the data collection tool based on the respondent’s responses. The data collection tool had closed ended questions on variables related to four main themes: the household, the housing unit, compound characteristics, and neighbourhood characteristics. A few variables in the housing unit such as materials used for construction were observed and recorded on the guide.

For quality and rigour, the research assistants were trained before data collection began. They were trained on aspects such as ethics in data collection, handling respondents, and presenting the questions to the respondents. The tools were first pretested and any questions that were not clear to both respondents and research assistants were revised.

### 3.3. Ethical Requirements

This study was approved by the Research Ethics Committee (REC) of Stellenbosch University, and we also obtained a permit from the Kenya National Commission for Science and Technology (NACOSTI) (No.: NACOSTI/P/14/5546/781). In addition to the permit, research authorisation letters were obtained from the Kisumu County Education office, and the chiefs within the settlements granted their permission before the data collection process began. Respondents were given full information, and allowed to give their consent before any interviews began. To ensure the anonymity of respondents, names and any personal identifiers were not used on the data collection tool.

### 3.4. Data Management and Analysis

At the end of data collection, data had been collected from 180 respondents. The data were entered into Epi-Info (Centre for Disease Control (CDC), Atlanta, GA, USA) and checked for any errors before transferring to Stata (Version 13) (Stata Corp, College Station, TX, USA) for analysis.

Based on principles of hedonic regression described in Section 2, the dependent variable was the amount of rent paid. The independent variables were grouped into four main themes/categories: in the housing unit theme, variables included duration of stay in a house, number of rooms, floor and wall construction material, and whether the house had electricity connection or not. Variables in the compound theme included; the number of houses, main water sources, travel time to water sources (in minutes), cost of water, presence of a sanitation facility, waste disposal methods, and type of residence. Neighbourhood characteristics included time taken to access the main road and access (link) road, and forms of transport used to access the Central Business District (CBD), workplace and nearby health centres. Household variables included individual characteristics such as age, education level, occupation, income levels and household size (summarised in Table 1). The assumption, as it is in hedonic pricing method, was that the amount of rent paid was a function of all these variables.

Analysis began with descriptive statistics to summarise and describe the dependent and independent variables. Histograms were used to assess the distribution of the variables for normality. Continuous variables were summarised through means, standard deviation and frequencies, while categorical variables were summarised through frequencies and percentages. Pearson’s correlation was used to check for linear relationships among pairs of each of the independent continuous variables, and chi square tests were used to assess associations among categorical variables.

Multiple logistic regression was further used to assess relationships between availability of sanitation, as the dependent variable, and the other independent variables. To estimate the effect of the independent variables on rent, multiple regression analysis was performed, in a stepwise manner, using linear, log-linear and double log regression models. Each of these models was assessed for its ability to predict the dependent variable by examining the value of the adjusted R-squared (R^2^).

Interaction between explanatory/independent variables was tested using the Wald test. The models were adjusted to account for heteroscedasticity by Huber/White’s/sandwich estimators of robust standard errors, and White’s general test for heteroscedasticity was applied. The variance inflation factor (VIF) was used to assess for multicollinearity among the independent variables. The model was tested for omitted variable bias using the Ramsey RESET test. All associations were tested at the 95% confidence level.

## 4. Results

Table 1 shows the descriptive summary of the study variables. Pearson’s correlation test showed weak linear relationships (r = 0.4 or less) between the continuous independent variables. Some associations were noted between the categorical variables, for example, most (71%) compounds without sanitation facilities had absentee landlords (Chi^2^ (2) = 24.89, *p* < 0.001), and respondents from compounds without sanitation facilities paid lower rent (Chi^2^ (3) = 22.19, *p* < 0.001).

In order to understand individual characteristics of respondents with sanitation facilities, logistic regression results indicated that the odds of having a toilet when one was married (compared to being unmarried or a single parent) was 4.6 times greater (*p* = 0.008), and when one had secondary education (compared to not having any education) it was 4.3 times greater (*p* = 0.02). These results are confirmed by cross-tabulation results, which indicate that 70% of the respondents who were single or single parents lived in compounds without sanitation facilities. In addition, these compounds with sanitation facilities also had better services, such as an electricity connection (Chi^2^ (1) = 14.2933, *p* < 0.01). These compounds also had better house construction materials for the walls and floors as it was noted that among respondents living in compounds with toilets, 71% had houses that had rough-cast walls (Chi^2^ (2) = 15.8975, *p* < 0.01).

Results of the multiple logistic regression revealed that for every one unit increase in rent (KES per month), the odds of having a toilet increased by 1% (*p* = 0.02, CI: 1.000–1.002) while for every increase in the number of households, the odds of having a toilet increased by 28% (*p* < 0.00, CI: 1.11–1.48). However, the odds of having a toilet reduced by 18% in compounds with absentee landlords compared to compounds with live-in landlords (*p* = 0.008, CI: 0.05–0.64).

Table 2 shows the coefficients, robust standard errors and P-values of variables in the linear, log-linear and double log models of the hedonic regression. The double log model was adopted, as it gave the best prediction and the highest value of R^2^ (55.1%). In this model, both the dependent and the independent variables were transformed to the natural log form.

Variables from the housing unit theme of independent variables explained approximately 43% of the variation in rent, compound characteristics explained 4.6%, area (settlement) characteristics explained 3.9%, household characteristics explained approximately 2.7%, and neighbourhood characteristics explained 0.5% of the variation in rent.

From Table 2 and based on the understanding that the hedonic pricing method assumes that the rent paid is a reflection of the willingness to pay for the attributes that make up the house, it is clear from the double log regression model that residents paid a higher amount of rent for housing that had more than one room, had an electricity connection, and had better walls and floors. At the compound level, residents in compounds with sanitation facilities paid a higher amount of rent. Sanitation constituted 54% ((e ^0.434^−1) × 100 = 54%) of the rent, implying that on average, it cost households KES 655 every month to live in compounds with sanitation facilities. The regression results showed a negative interaction effect between having a toilet with an increase in the number of households. From the coefficient of the interaction (−0.155), rent reduced by 16% for every integer increase in the number of households sharing toilets.

Overall, the regression model gave a mean variance inflation factor of 1.73, thus an indication that the independent variables were not linear combinations of each other. In addition, White’s test for heteroscedasticity gave a chi square value of 143.6, with a *p*-value of 0.49, which led to accepting the null hypothesis that there was equal variation among the independent variables, hence no heteroscedasticity. The Ramsey RESET test for omitted variables led to the acceptance of the null hypothesis that the model had no omitted variables (F (3, 144) = 1.68, *p* = 0.17), thus leading to the conclusion that more variables are not needed to predict the dependent variable.

Informal interviews with community residents and leaders indicated that, on average, it cost approximately KES 60,000 to construct a simple pit latrine with brick walls, iron sheet roofing and cemented floor slab. A landlord would therefore recoup KES 655 per month for sanitation with one tenant household, and it would take approximately 91.6 months to fully recover the investment costs of the sanitation facility. On the other hand, if a landlord had seven tenant household (the average number of households per compound in this study), he would recoup KES 4585 per month, and it would take approximately 13 months to recover the amount he invested in sanitation facilities.

**Figure 1:**
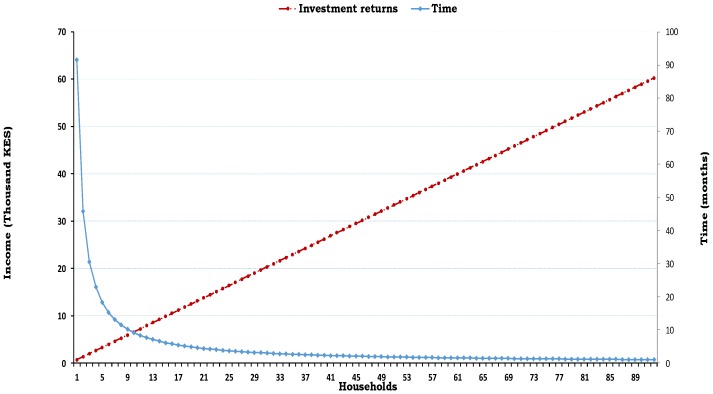
Projection of time taken to recover investment in sanitation against number of households (these projections are based on a single pit latrine, and they exclude any extra expenses, such as repair and emptying costs).

Figure 1 is a projected estimate of the time it would take a landlord to recoup sanitation investment costs against the number of tenant households in a compound.

## 5. Discussion

### 5.1. Estimating Cost of Sanitation through the HPM

Knowledge of the cost and value of sanitation is critical, especially in poor urban areas. Studies have used the contingent valuation approach, with very few taking on a hedonic approach to estimate the cost of sanitation in informal settlements. This study adopted a hedonic approach and compared the linear, semi-linear and double log models to estimate the effect of sanitation on rent. Although the three models gave varying effects of sanitation on rental prices, the values of R^2^ from each of the three models were not very different, and the effect of sanitation was significant in all three models.

The findings further revealed the association between having sanitation facilities and better quality housing, and also that the availability of sanitation facilities constituted a substantial amount of rental prices. These findings concur with those of a study by Gulyani et al. [45], who used a hedonic approach with a log linear model to assess the determinants of rent prices in the informal settlements of Dakar (Senegal) and Nairobi (Kenya). They noted that access to a toilet (shared by 10 households or less) in Nairobi’s informal settlements raised the monthly rent by 1.6%. These percentages in Gulyani’s study are substantially lower than those in this study, and this may be attributed partly to regional differences. For example, the study by Gulyani et al. [45] included several informal settlements in each of the two cities, which may also have different socio-economic conditions and preferences. Kibera in Nairobi, for instance, has had a number of interventions of communal sanitation facilities, with a study by Schouten and Mathenge [56] indicating that the residents preferred communal sanitation alternatives. With such preferences, it is likely that their willingness to pay a higher amount of rent for sanitation facilities that are shared by a number of households may be lower.

Other hedonic studies have highlighted increments in rental values due to sanitation, for example an increment of 11.5% to 32% in Bangladesh [57], a 20% increment in Togo [58], and a 60% increment in Sri Lanka [26]. A few others have highlighted the incremental effect of “improved” sanitation facilities and technologies. From Indonesia, Brueckner [46] highlights that housing properties with their own toilets had a rent 14% higher compared to housing structures that lacked sanitation facilities or that had shared sanitation facilities. In Uganda, Knight et al. [59] highlighted that flush toilets increased the rent by 42%, while a pit latrine led to a 26% increase in rent. Similarly, in Nigeria, Ajide and Kareem [60] report that an improved technology (such as a flush toilet) attracted a higher increase in rent than an unimproved sanitation facility such as a pit latrine or a bucket latrine. The same findings are also expressed by Jenkins et al.’s [61] study in Tanzania, in which it was reported that households with improved sanitation facilities paid higher rents.

Although the type of sanitation technology is an important consideration, this study did not investigate the effect of different sanitation technologies, since all the respondents used pit latrines. The prevalent use of pit latrines is not surprising, since other studies and reports indicate that there are fewer sanitation technologies within Kisumu’s informal settlements, and that pit latrines are used by the majority, while septic tanks serve a small minority [48,52,62]. This predominance of pit latrines is common in other informal settlements in Uganda [63], Tanzania [9,13], Rwanda [64], Senegal [32] and Ghana [65]. Possible users of the few septic tanks in Kisumu’s informal settlements would most likely be home owners, who were purposely left out of this study because they were not rent payers.

### 5.2. Economics of Sanitation in the Complex Dynamics of Informal Settlements

In order to understand the economic dynamics of sanitation in Kisumu’s informal settlements, it is necessary to explore other factors that directly or indirectly influence payment for sanitation. One of these is the characteristics of residents with sanitation facilities. The results of the logistic regression reveal that residents who were likely to have sanitation facilities were those who had secondary education and were married. Education is not only important in urban informal settlements, but in the rural areas too, as confirmed by studies from Tanzania [66] and India [67], which found that educated individuals were more likely to own and use sanitation facilities. Similarly, in Indonesia, it was noted that individuals with higher levels of education were likely to select housing with better characteristics, such as toilets, hence they paid more for rent [46]. These findings confirm that educated households are knowledgeable about the importance of sanitation, they choose to live in compounds with sanitation facilities, and therefore are willing to pay a higher rent in order to acquire sanitation.

Another important characteristic is income, since it is expected that income determines the purchase of sanitation facilities or the paying of a higher amount of rent (according to neoclassical economics). The results from the logistic and hedonic regression models, however, suggest otherwise. Similar findings are reported from rural Tanzania, where Sara and Graham [66] found that income was not a significant factor for acquiring and using toilets. With this contrast, it becomes imperative to understand if income is a barrier to or determinant of the acquisition of sanitation in informal settlements. A study from informal settlements in Kampala, Kisumu and Kigali [68] highlighted that “inability to afford” improved sanitation hindered demand for sanitation in the informal settlements. In contrast, the findings from this study suggest that affordability is just but one determinant. Although high costs can lock out the poor, who may not be able to afford sanitation, there are other factors that also explain payment for and acquisition of sanitation facilities in informal settlements aside from income.

The results of the multivariate logistic regression gave an indication of some of these factors. The results revealed that individuals living in compounds with more households were likely to have toilets, while those in compounds with absentee landlords were less likely to have sanitation facilities. It is within these “compound” factors that complexities of payment for sanitation within informal settlements are hidden. Some of these complexities can be explained by land tenure factors, which Scott et al. [32] highlight as being crucial because they greatly influence investment in sanitation in informal settlements.

To examine the effect of tenancy, the results indicated that tenants living in compounds with absentee landlords were more likely to pay lower rents. This finding can be linked to the study by Okurut and Charles [68], who highlighted that the main hindrance to installing sanitation facilities in Kisumu was a lack of space, because most of the available space had been used to construct rental housing units. The explanation for these findings is that landlords, especially absentee landlords, are more likely to focus on constructing housing units (which may not be of good quality because the rents are low) so that they can maximise returns in the form of rent. For such landlords, constructing sanitation facilities may be costlier (and without immediate returns) than constructing housing units (which have monthly returns), hence the reason why low-quality housing units often lack sanitation facilities. It therefore becomes crucial to understand the cost of investing in sanitation.

The estimated cost of investment in a single pit latrine found in this study falls within the range of costs quoted in Uganda, of approximately USD 418 to 1250 [13,37], but is higher than the range in Tanzania, of approximately USD 200 to 445 [13,61]. The projection in Figure 1 (Figure 1 is meant to serve as a demonstration and projection of the length of time taken to recoup investments in a sanitation facility against the number of household tenants) suggests that landlords are likely to recover their investment in a shorter time if more households share sanitation facilities. The results also show that compounds with more households were more likely to have sanitation facilities, but the rent decreased with increasing number of households. These results suggest that landlords, who often times constructed the facilities themselves or hired local community masons who would often times be unskilled [37,62] can spread the costs of investment in sanitation among many households, which implies that the cost per household may be substantially lower compared to the cost per household in a compound with fewer households.

Landlords therefore have to make decisions on whether to provide sanitation facilities shared by fewer tenants who pay slightly higher rents, or have more tenants sharing a toilet and paying slightly lower rent. These projections, however, exclude other expenses that a landlord may incur that are common to pit latrines, namely operation and maintenance in the form of emptying and repairs. The frequency of pit latrine emptying is determined by factors such as number of users/loading rate, size of the pit, the type of materials dumped into the pit (materials like plastics and sanitary pads make the pits fill up faster due to the long time it takes for them to decompose) and the level of the water table, especially during rainy/flooding seasons [13,69]. Depending on these factors, the frequency of emptying varies, with studies indicating that some pits are emptied as often as every one to six months [13,69], while others are emptied only after a couple of years [70] (It was not possible to estimate the pit latrine emptying frequency because of differences in recall, and also because some tenant respondents had not had their pit latrines emptied). The cost of emptying varies, with preliminary studies [71] and informal interviews showing that households in the settlements prefer manual pit latrine emptiers, who charge a negotiable rate of KES 3000 to 6000. A landlord with many household tenants may recoup his investments faster from rent, but some of it may be used for the operation and maintenance of the pit latrines. Similarly, a landlord with fewer tenants may take a longer time to recover his investment, but would also spend less on operation and maintenance. These economic dynamics partly explain why some landlords do not provide sanitation facilities and some are less concerned about operation and maintenance. As a result, some tenants used toilets in neighbouring compounds, which then led to high loading rates of the pit latrines. To deal with some of these challenges, some compounds had their toilets locked to keep out members from other compounds, while some live-in landlords allowed tenants from other compounds to use their toilets, but with additional charges.

Landlords may also be less motivated to construct sanitation facilities if they have fewer households in a compound because it is easier to have fewer tenants finding alternatives (such as sharing sanitation facilities with their neighbours) rather than a higher number of households. For a landlord, it is a “safer risk” not to provide sanitation facilities to a smaller number of tenants than to a greater number of tenants. Tenants, on the other hand, can opt to live in compounds without sanitation facilities and with low-quality housing where they pay lower amounts of rent because they can share sanitation facilities with their neighbours without any (or with minimal) payment, especially if they have good neighbourly relations.

A second (social) explanation for the low rent in compounds with a higher number of households is related to the “free riding” phenomenon experienced with shared goods. When sanitation facilities are shared by many households, some users may not participate in maintenance practices such as cleaning, or some may not be willing to take responsibility for a shared facility. As a result, shared sanitation facilities may not be maintained as properly as private facilities would, rendering them dirty and unpleasant to use. Dirty, shared sanitation facilities leads to dissatisfaction among users, as has been reported in informal settlements in Uganda [63] and Rwanda [64]. Tenants would therefore not be willing to pay higher rental values for poor-quality shared sanitation facilities with which they are not satisfied, as was also noted in Bangladesh [57]. These findings suggest that tenants in informal settlements prefer private household sanitation facilities, or facilities that are shared by fewer households.

These socio-economic dynamics therefore reveal that there are differences between landlords’ and tenants’ preferences, thus advancing the theory that tenants have less incentive to invest in sanitation facilities because landlords will harness the benefits through rent increments [8,72]. These different preferences have also been reported in studies done in Kampala and Dar es Salaam [9], as well as in Ghana [65]. Tenants may not be willing to invest in sanitation because of their “temporary” status, and because they feel it is the landlord’s responsibility. Landlords, on the other hand, may have reasons to increase rental prices if they provide sanitation facilities. Okurut and Charles’s [68] study further reported that tenants in the informal settlements showed no demand for sanitation (by showing no indication of preference to install a sanitation facility). The reason why preference (or demand for sanitation) seemed to be low in Okurut and Charles’s study is due to the temporary or insecure tenure status of tenants. The findings from the current study point to a different conclusion, namely that there is a demand for sanitation (services and technologies), albeit through higher rental prices. The findings suggest that the residents of Kisumu’s informal settlements may be willing to pay for sanitation, and some are actually paying for sanitation. In the same manner, Ahmad [57] found that urban residents in Bangladesh (irrespective of whether they were tenants, owners or squatters) had a demand for sanitation facilities.

Finally, aside from the socio-economic factors discussed, there may be other factors, such as social and cultural norms, which explain payment for sanitation but which may not be explained by economic models. To illustrate the limitation of economic models, O’Keefe et al. [73] argue that, even though an investment in sanitation or a behaviour change could lead to an improvement, it is not sufficient to assume a perfectly rational assessment in sanitation decision making, since an individual might make contrary decisions because of social and cultural norms. In the same manner, although from a study in rural India, London et al. [74] arrive at a similar conclusion that economic factors are not the sole reasons driving consumer purchase decisions, and that social norms are equally influential. Economic models may be a pointer to the cost of sanitation in informal settlements, but other factors that cannot be measured directly but that play a crucial role in influencing decisions about sanitation, such as social and cultural norms, are equally critical. Such factors may be embedded within the complex social dynamics in the settlements and require the use of multiple research approaches and the involvement of various stakeholders within the settlements.

## 6. Implications for Policy and Areas for Further Research

The findings of this study have an implication for both sanitation promoters and policy makers. The cost of sanitation as reflected in the rental prices shows that tenants in informal settlements would benefit greatly from sanitation, but cost is a major factor that limits sanitation acquisition. For promoters it is crucial to identify affordable sanitation technologies and determine a minimum cost that will not lock out the poor. After the identification of an appropriate sanitation technology, policy makers need to liaise with landlords and tenants in informal settlements and identify strategies that will increase sanitation provision in informal settlements, especially for those who may not be able to afford sanitation. Possible avenues for ensuring access could include subsidising the cost of sanitation or providing opportunities for access to finances for installing sanitation facilities. Subsidies/finances could be in the form of construction materials provided to landlords as loans. Such financial approaches, however, should have adequate monitoring and repayment strategies. It is also necessary that approaches involve training the semi-skilled individuals in the settlements so that they can improve on the services they offer.

For further research it is necessary to identify and test possible sanitation technologies, as well as to estimate their costs. Such estimation should include other expenses that are likely to be incurred, such as costs of repair and faecal sludge management.

### Limitations

As mentioned in the discussion, this study was carried out among the rent paying residents in Kisumu’s informal settlements. The most dominant sanitation technology was the pit latrine, and other technologies were not considered.

## 7. Conclusions

This study has investigated the urban poor’s payment for sanitation in the informal settlements of Kisumu, Kenya through their revealed preferences. Using the hedonic pricing method which estimates the willingness to pay for housing attributes through the rental amounts, the findings show that the urban poor are willing to pay for sanitation since those living in compounds with sanitation facilities pay a significantly higher amount of rent. However, residents of informal settlements are faced with a number of limitations and payment for sanitation is intertwined in the complex dynamics within informal settlements. Factors influencing payment for sanitation are related to individual factors such as education, as well as compound factors such as land tenure and the sharing of sanitation facilities. Tenants are willing to pay for better sanitation services, and landlords stand to benefit by investing in quality housing with good quality sanitation facilities that are well maintained by the users. Other dynamics within informal settlements, such as norms and relations between residents, also play a role in influencing payment for sanitation, and it is important that they are taken into consideration when planning sanitation interventions.

## Figures and Tables

**Table 1:** Summary of study variables (*n* = 180, unless stated otherwise).

Variables		Mean (Range)/Freq (%)
1. Household	Age	30.36 (18–65)
	HH size	3.88 (1–9)
	*Gender*	
	Male	33 (18.3)
	Female	147 (81.7)
	*Education*	
	None	61 (33.9)
	Primary education	97 (53.9)
	Secondary education and above	22 (12.2)
	*Marital status*	
	Single/unmarried/single parent	24 (13.3)
	Married	128 (71.1)
	Widowed/divorced/separated	28 (15.6)
	*Occupation*	
	None/housewife	65 (36.1)
	Casual worker	33 (18.3)
	Self-employed/business	76 (42.2)
	Formal employment	6 (3.3)
	Monthly household income *	10,588.76 (0–90,000)
2. Housing unit	*Electricity*	
	Connected	78 (43.2)
	Not connected	102 (56.7)
	*Wall*	
	Mud	62 (34.4)
	Iron sheet	16 (8.9)
	Plastering/Rough cast	102 (56.7)
	*Floor*	
	Mud	52 (28.9)
	Cemented/concrete	128 (71.1)
	*No. of rooms*	
	1	139 (77.2)
	2	34 (8.9)
	3	7 (3.9)
	Rent *	1211.7 (300–3500)
	*Area*	
	Bandani	40 (22.2)
	Nyalenda A	47 (26.1)
	Nyalenda B	50 (27.8)
	Obunga	43 (23.9)
3. Compound	*Number of HH*	7 (1–25)
	*Main water source*	
	Compound connection	14 (7.8)
	Nearby water point	148 (82.2)
	Neighbour’s compound	14 (7.8)
	Others	4 (2.2)
	*Time to walk to water source (in minutes)*	
	Compound connection	14 (7.8)
	Less than 5 min	111 (61.7)
	5 min and above	55 (30.6)
	*Cost of water at main source* (20 litre jerry can) *	3.2 (1–5)
	*Residence type*	
	Live-in landlord	45 (25)
	Tenants with caretaker	40 (22.2)
	Tenants only	95 (52.8)
	*Sanitation*	
	Available	91 (50.6)
	Not available	89 (49.4)
4. Neighbourhood	Time to main road (minutes)	14.5 (1–60)
	*Transport to work place* *(n = 115)*	
	Walking	92 (80)
	Bicycle/motorbike	12 (10.4)
	Three-wheeler cars/minibus	11 (9.6)
	*Time to workplace* *(minutes)*	15.8 (1–120)
	*Transport to city centre*	
	Walking	50 (27.8)
	Bicycle/motorbike	53 (29.4)
	Three-wheeler cars/minibus	77 (42.8)
	*Time to city centre (minutes)*	28.1 (5–120)
	*Transport to health facility*	
	Walking	110 (61.1)
	Bicycle/motorbike	35 (19.4)
	Three-wheeler cars/minibus	35 (19.4)
	*Time to health facility (minutes)*	(0–60)

* Amounts in Kenyan Shillings (KES). 1 United States Dollar (USD) = 100 Kenyan Shillings.

**Table 2:** Results of the linear, log-linear and double log regression models of determinants of rent prices in informal settlements of Kisumu.

Variables	Log-Log			Log-Linear			Linear		
	Co-Eff	S.E. **	*p* Value (CI)	Co-Eff	S.E. **	*p* Value (CI)	Co-Eff	S.E. **	*p* Value (CI)
Electricity	0.234	0.066	0.001 (0.103–0.365) *	0.209	0.066	0.002 (0.077–0.341)	325.208	94.767	0.001 (137.94–512.47)
Iron sheet wall	0.130	0.105	0.217 (−0.077–0.339)	0.176	0.104	0.094 (−0.031–0.384)	143.591	115.357	0.215 (−84.35–71.538)
Rough-cast wall	0.182	0.075	0.018 (0.032–0.332) *	0.233	0.076	0.003 (0.082–0.383)	262.763	83.762	0.002 (97.24–28.278)
Two-roomed house	0.330	0.076	0.000 (0.179–0.481) *	0.305	0.071	0.000 (0.164–0.448)	389.89	91.909	0.000 (208.27–571.505)
Three-roomed house	0.503	0.130	0.000 (0.246–0.761) *	0.503	0.126	0.000 (0.253–0.753)	869.301	277.880	0.002 (320.21–1418.39)
Cemented floor	0.166	0.071	0.021 (0.025–0.307) *	0.107	0.072	0.14 (−0.035–0.251)	102.624	82.720	0.217 (−60.831–266.08)
Nyalenda A	0.090	0.077	0.242 (−0.061–0.242)	0.103	0.073	0.161 (−0.042–0.249)	107.564	89.055	0.229 (−68.410–283.54)
Nyalenda B	0.235	0.080	0.004 (0.076–0.394) *	0.233	0.073	0.002 (0.088–0.378)	210.048	88.291	0.019 (35.583–384.51)
Obunga	0.156	0.087	0.074 (−0.015–0.328)	0.155	0.084	0.068 (−0.012–0.323)	98.996	103.03	0.338 (−104.6–302.5)
Toilet	0.434	0.170	0.012 (0.097–0.772) *	0.342	0.129	0.009 (0.086–0.598)	418.252	172.1	0.016 (78.18–758.32)
Compound HH	0.060	0.067	0.368 (−0.072–0.193)	0.011	0.014	0.44 (−0.017–0.039)	13.761	16.88	0.416 (−19.59–47.11)
Toilet#comp HH ^a^	−0.155	0.086	0.076 (−0.326–0.016)	−0.025	0.015	0.112 (−0.057–0.005)	−39.190	19.761	0.049 (−78.24–−0.141)
Nearby water point	−0.020	0.106	0.851 (−0.231–0.190)	−0.020	0.108	0.847 (−0.236–0.194)	−65.266	167.13	0.697 (−395.52–264.98)
Neighbours water	0.0423	0.134	0.753 (−0.223–0.307)	0.015	0.132	0.908 (−0.247–0.278)	−9.8276	202.71	0.961 (−410.4–90.747)
Others-borehole, springs)	0.190	0.129	0.142 (−0.064–0.445)	0.229	0.130	0.081(−0.028–0.488)	183.851	187.605	0.329 (−186.86–554.56)
Time to main road	−0.027	0.034	0.421 (−0.096–0.040)	−0.001	0.002	0.465 (−0.007–0.003)	−2.1150	3.4621	0.542 (−8.95–4.72)
Primary educ	0.078	0.055	0.163 (−0.032–0.188)	0.068	0.052	0.193 (−0.035–0.172)	45.081	62.134	0.469 (−77.69–167.86)
Secondary educ	0.237	0.093	0.012 (0.052–0.42) *	0.211	0.093	0.025 (0.026–0.395)	293.16	144.79	0.045 (7.04–579.28)
Monthly income	0.024	0.033	0.466 (−0.041–0.090)	4.48	2.62	0.089 (−6.86–9.66)	0.00939	0.003	0.012 (0.002–0.016)
N			167			169			169
F statistic			12.69			13.11			10.14
*p*			0.000			0.000			0.000
R^2^			0.551			0.548			0.546

^a^ Test of interaction between having a toilet and number of households in a compound; * Significant at the 95% confidence level; ** Robust standard errors. Co-Eff: coefficient; S.E.: standard error (robust); CI: Confidence Intervals.
